# Supplementary material for: Disease decreases variation in host community structure in an old-field grassland
Source: PLoS One. 2023 Oct 27;18(10):e0293495. doi: 10.1371/journal.pone.0293495 (PMC10610459; doi:10.1371/journal.pone.0293495)
Supplement: S1 Table — Reported are % change in AUDPS relative to the control, bootstrapped LRR and 95% confidence intervals. (DOCX) [file pone.0293495.s001.docx]

| **Year** | **Treatment** | **% change** | **LRR** | **95% CI** | |
| --- | --- | --- | --- | --- | --- |
| 2017 | 7 months | -12.5 | -0.13 | (-0.15, | -0.11) |
|  | 9 months | -28.5 | -0.34 | (-0.40, | -0.27) |
|  | year-round | -42.4 | -0.55 | (-0.62, | -0.49) |
| 2018 | 7 months | -13.9 | -0.15 | (-0.18, | -0.12) |
|  | 9 months | -27.9 | -0.33 | (-0.42, | -0.23) |
|  | year-round | -35.6 | -0.44 | (-0.52, | -0.36) |
| 2019 | 7 months | -63.3 | -1.00 | (-1.16, | -0.85) |
|  | 9 months | -82.5 | -1.74 | (-2.03, | -1.44) |
|  | year-round | -85.9 | -1.96 | (-2.22, | -1.72) |
