# Supplementary figures and images for: Disease decreases variation in host community structure in an old-field grassland
Source: PLoS One. 2023 Oct 27;18(10):e0293495. doi: 10.1371/journal.pone.0293495 (PMC10610459; doi:10.1371/journal.pone.0293495)

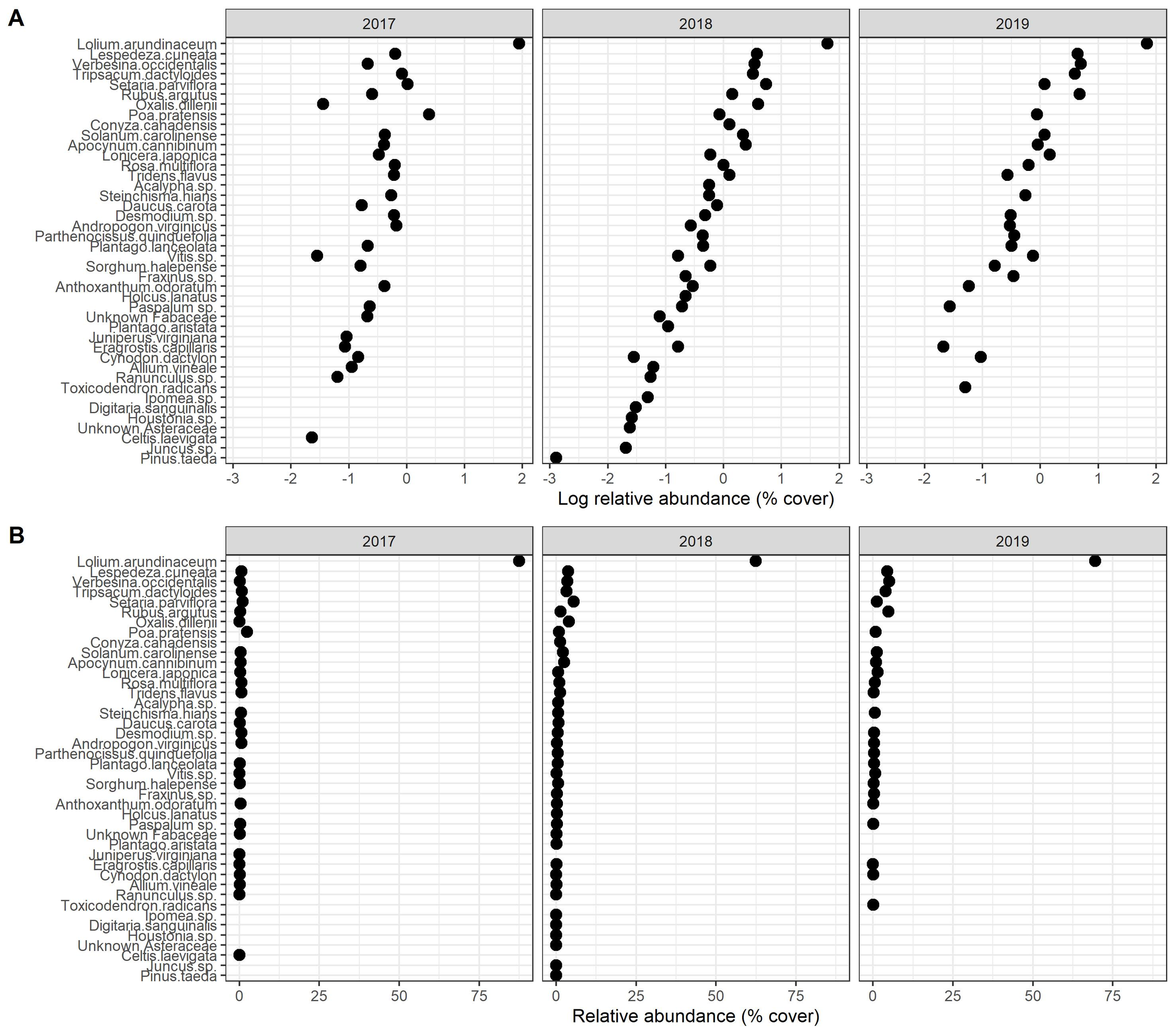

Supplement: S1 Fig — Relative abundance (% cover) was averaged across all 64 experimental plots for a given year. Tall fescue (Lolium arundinaceum) was the dominant plant species each year. (TIF) [file pone.0293495.s002.tif]

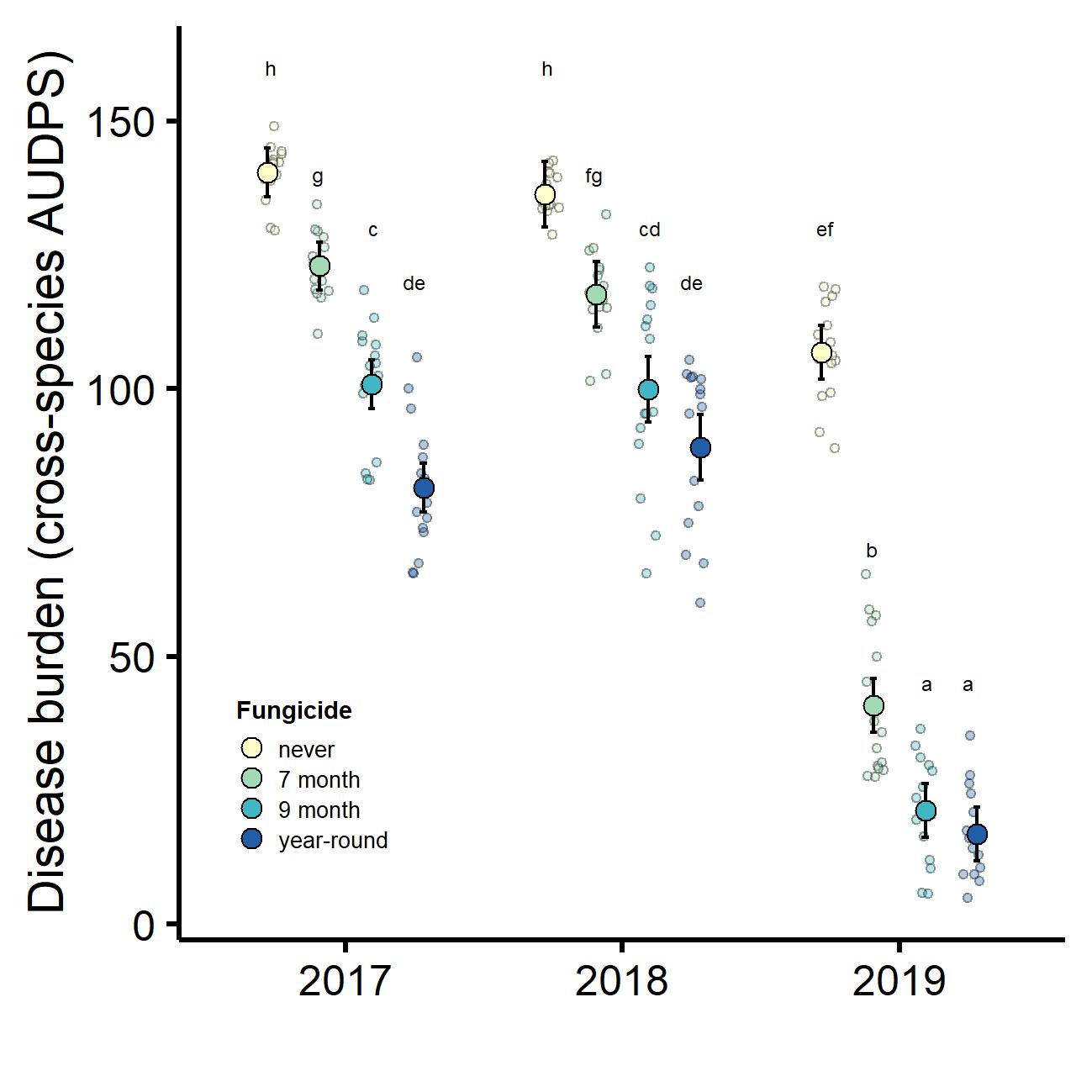

Supplement: S2 Fig — Plotted are observed treatment means and their 95% confidence intervals, and smaller points represent the raw data that are jittered to show the distribution of the data. Lower case letters denote groups based on Tukey post-hoc tests using all pairwise comparisons to compare treatment effects over time. (TIF) [file pone.0293495.s003.tif]

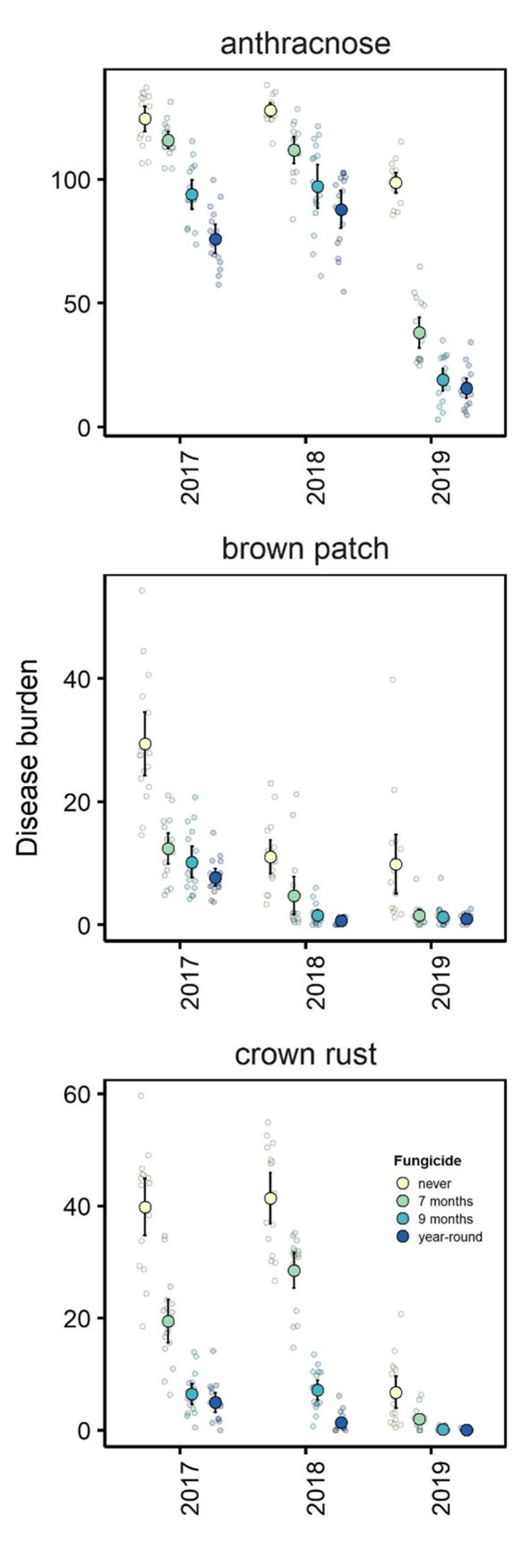

Supplement: S3 Fig — Large points indicate treatment means with their 95% confidence intervals, while smaller points are the raw data. Raw data points are jittered to show the distribution of the data. (TIF) [file pone.0293495.s004.tif]

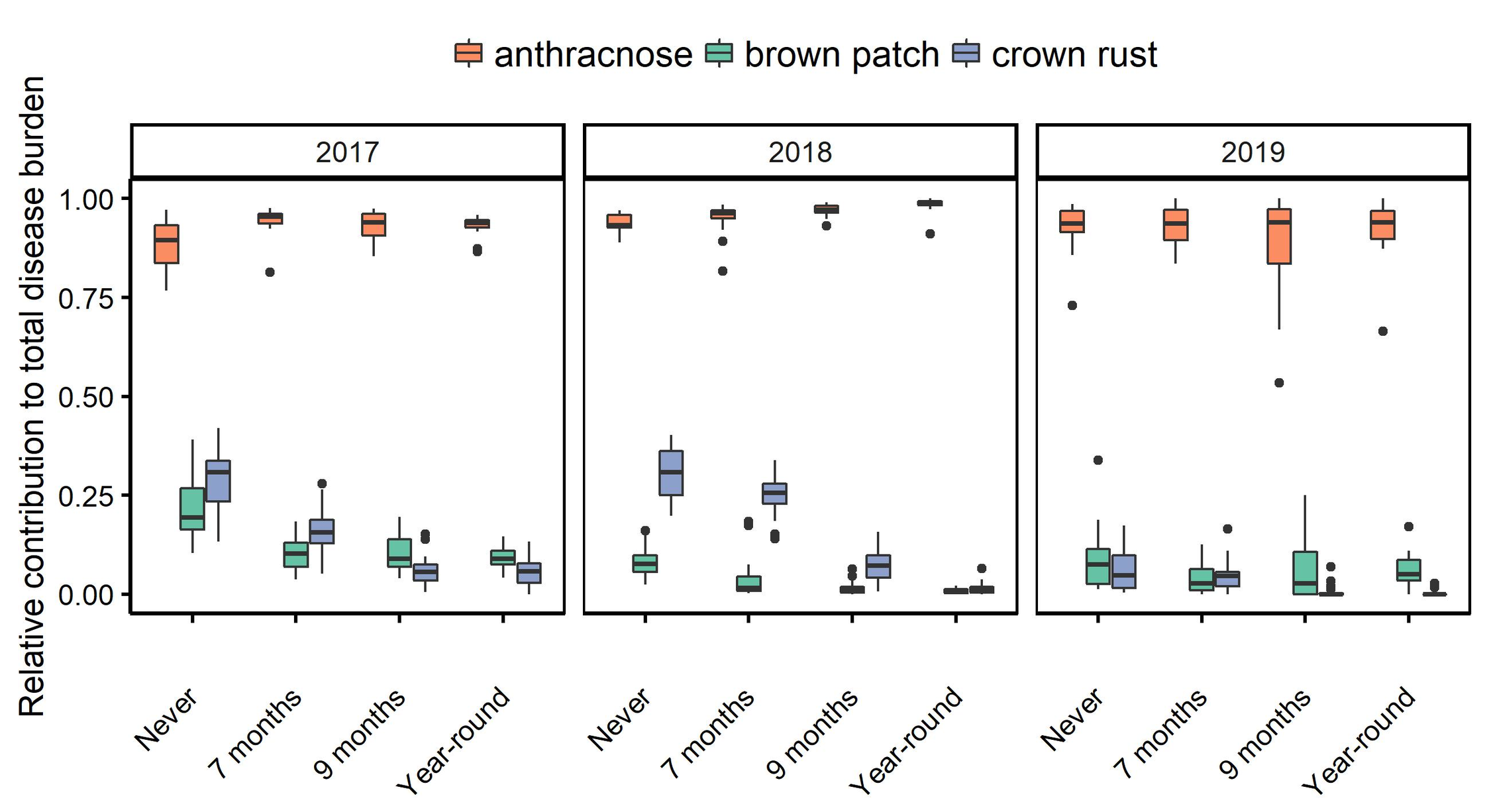

Supplement: S4 Fig — The disease anthracnose was the chief contributor to total disease across all treatments and years. (TIF) [file pone.0293495.s005.tif]

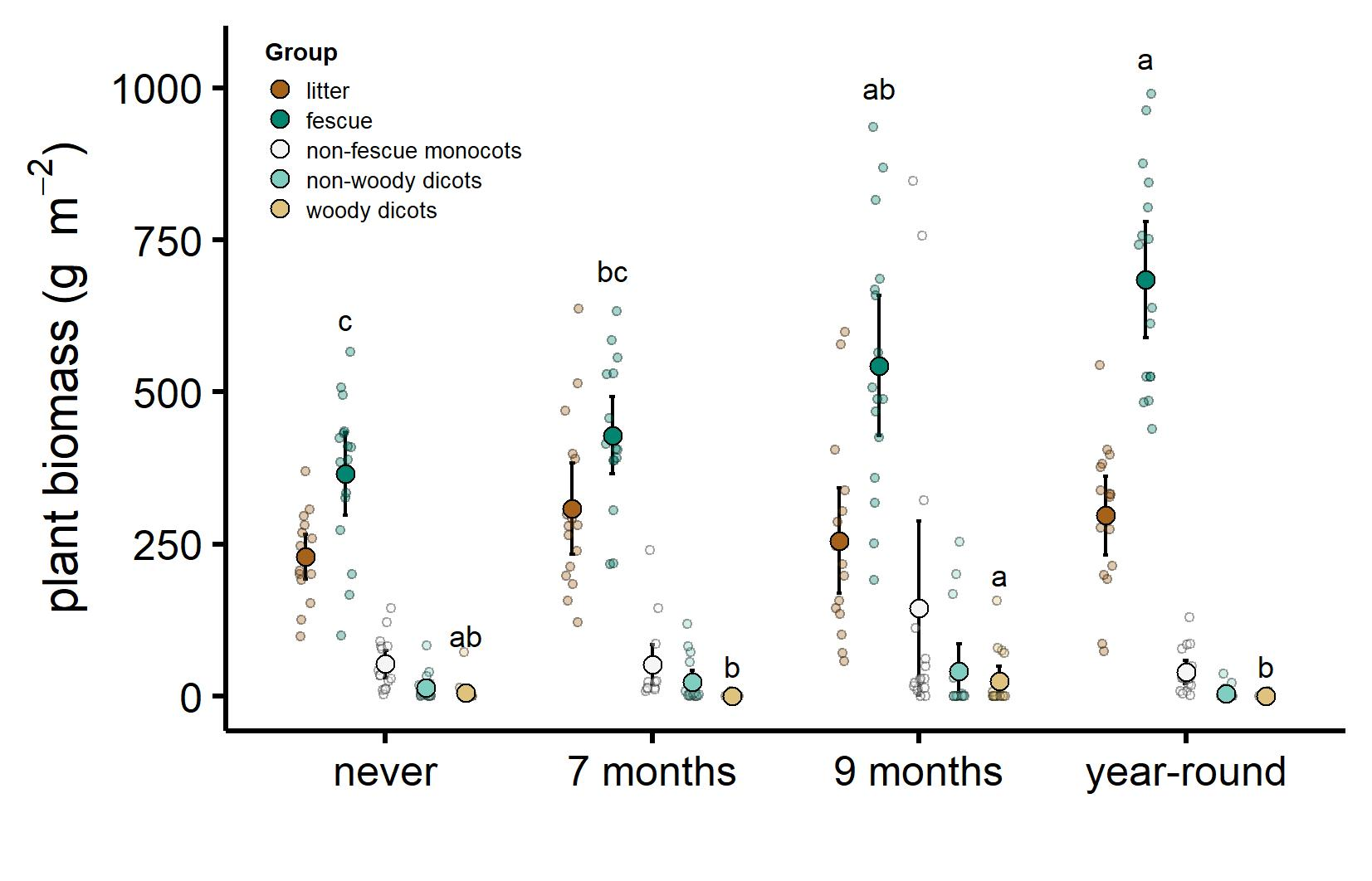

Supplement: S5 Fig — Specifically, differences among treatments in 2018 plant biomass were attributed to tall fescue (ANOVA, treatment: F3,60 = 11.47, p <0.0001, η2 = 0.36) and woody dicots (F3,60 = 3.32, p = 0.03, η2 = 0.14). Litter (F3,60 = 1.32, p = 0.28), non-fescue monocots (F3,60 = 1.87, p = 0.14), and non-woody dicots (F3,60 = 1.72, p = 0.17) were not affected by fungicide treatments in 2018. Letters denote post hoc comparisons between treatments within each plant biomass group. Plotted are observed treatment means and their associated 95% CI, and smaller points represent the raw data that are jittered to show the distribution of the data. (TIF) [file pone.0293495.s006.tif]

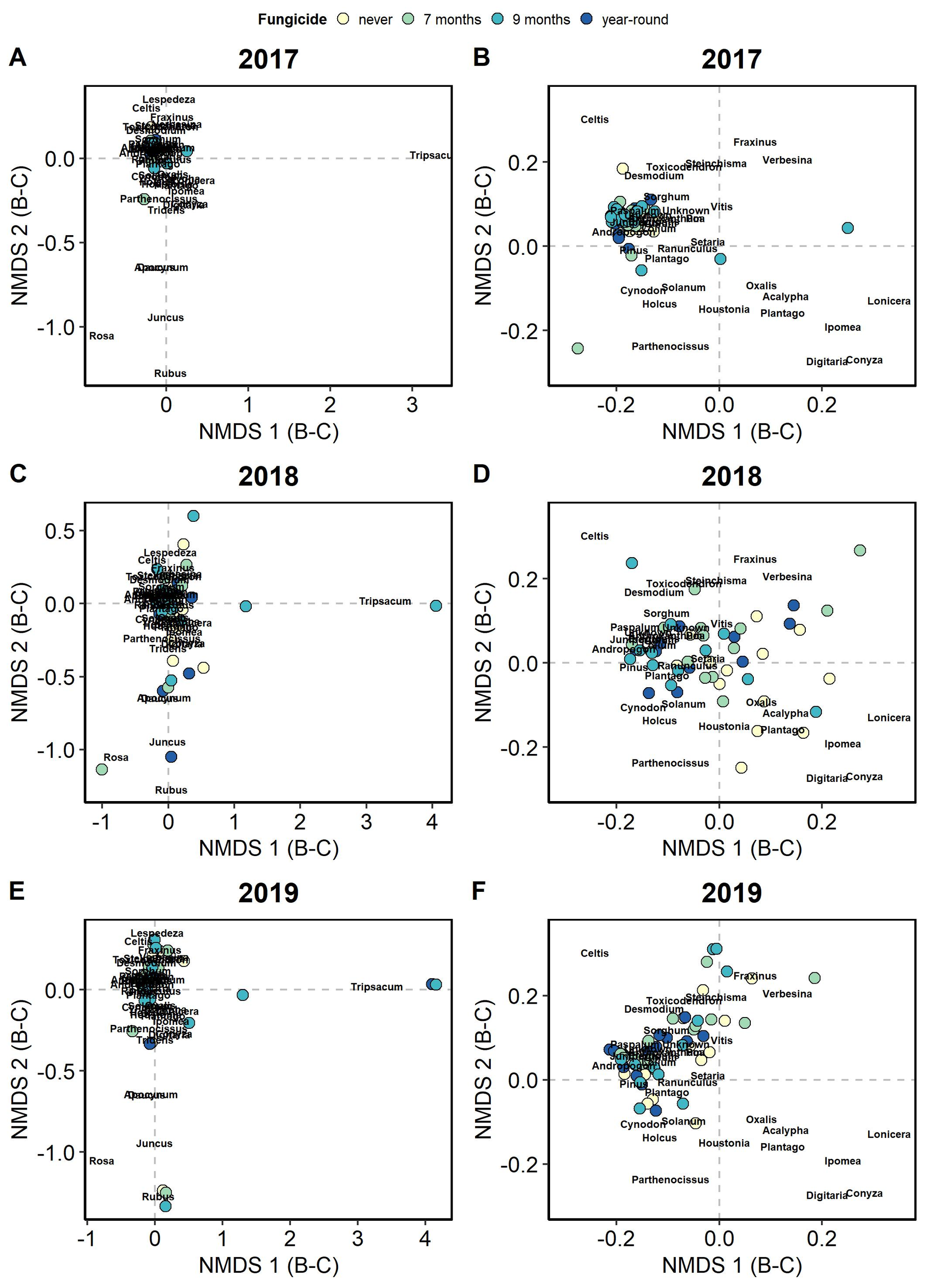

Supplement: S6 Fig — Shown are results of nonmetric multidimensional scaling of plant communities based on Bray-Curtis distances. All data are plotted on the same axes for A, C, E to show all community dissimilarity patterns. Panels B, D, F, are zoomed in to show the core community patterns. Species vectors show the distribution of plant taxa among communities. NMDS Stress = 0.12. (TIF) [file pone.0293495.s007.tif]

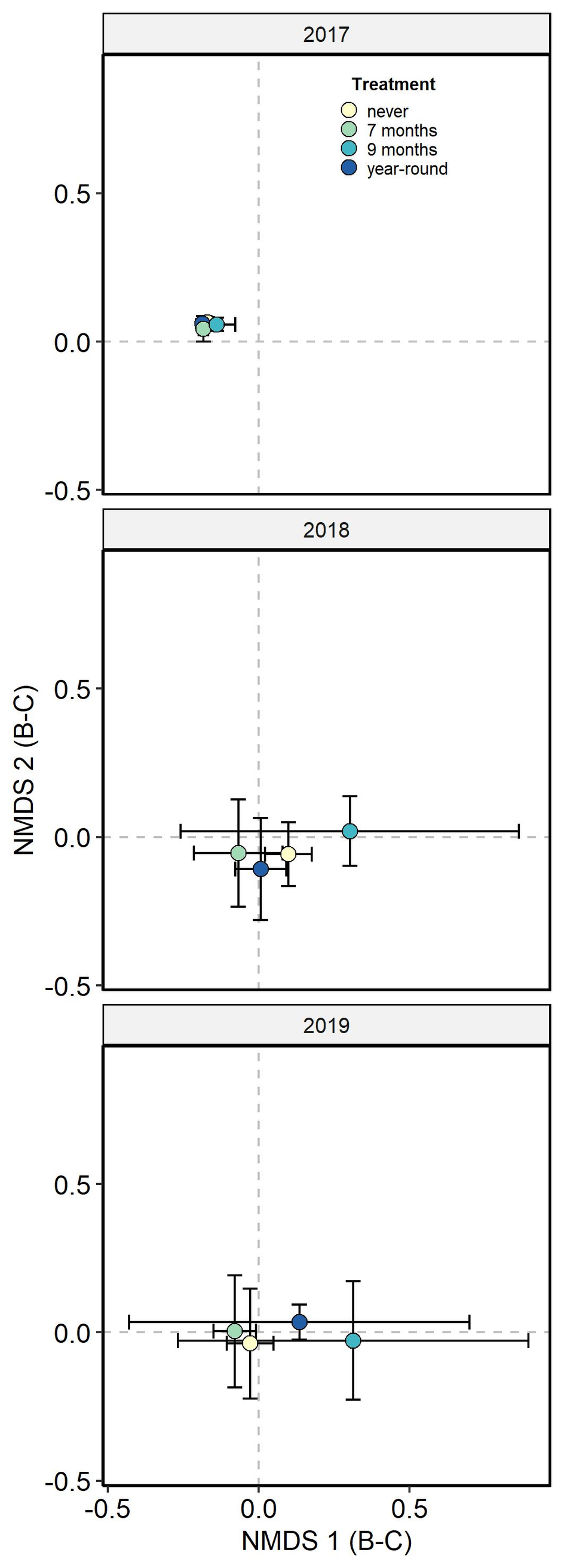

Supplement: S7 Fig — Plotted are treatment centroids of nonmetric multidimensional scaling based on Bray-Curtis distances and their 95% confidence intervals. NMDS Stress = 0.12. (TIF) [file pone.0293495.s008.tif]
