# Supplementary material for: Disease decreases variation in host community structure in an old-field grassland
Source: PLoS One. 2023 Oct 27;18(10):e0293495. doi: 10.1371/journal.pone.0293495 (PMC10610459; doi:10.1371/journal.pone.0293495)
Supplement: Attachment — Submitted filename: PlantCommunitySSE_ResponseLetter_R2_FINAL.docx [file pone.0293495.s010.docx]

Dear Editor,

Thank you for the opportunity to submit a revised version of our manuscript titled “Disease decreases variation in host community structure in an old-field grassland.” We thank the reviewers for their helpful comments and have revised the manuscript in response to their feedback. In response to reviewer 1, we have clarified parts of the introduction to better differentiate the hypotheses in the manuscript. We have provided a response letter to the comment from the reviewer with line references below in ***bold italics*** and a tracked changes version of our manuscript.

We feel these changes have improved the quality of our manuscript, and we hope that you will now find this work suitable for *PLOS ONE*. Thank you for your efforts in the publication of our research.

Sincerely,

Rita Grunberg

**Review Comments to the Author**

Reviewer #1: The authors successfully addressed almost all of my comments and I appreciated their responses. However, I still had to read the introduction a few times to understand the motivating theory/hypotheses. I know I already gave a lengthy comment on this in my last review, so I will try to explain it differently this time. I think that all the ideas are there but are difficult to understand due to unbalanced presentation of hypotheses and implicit assumptions. It also might help to reconsider the order of ideas in the introduction. The two potential outcomes of disease on convergence are presented in an unbalanced way because one is the main conceptual hypothesis and the other is system-specific hypothesis. The “main conceptual hypothesis” is presented on L23-24, L56-58, and L73-75: disease drives convergence of communities because species that are more tolerant/resistant become more dominant. This hypothesis needs more citations and/or explanation of implicit assumptions because it is unclear why the more tolerant/resistant species would be the same across communities (for example, if the disease is caused by a specialist parasite, there could be a whole range of tolerant/resistant species). Even if the tolerant/resistant species are the same across communities, the species impacted by disease could be more affected by stochastic processes as they experience symptoms or their populations decline. Note that I’m not asking you to put these specific alternatives in the text. My aim is to demonstrate why it’s not intuitive to me that this should be the expected outcome over other outcomes. The “system-specific hypothesis” is presented on L106-110 and hinted on L425-428: reducing disease with fungicide drives convergence of communities because more impacted species become more dominant. This hypothesis is subject to the same question as the first hypothesis.

***Author: Thank you for the clarification. In the introduction, we have included more references in certain parts to support our hypotheses. In addition, we added text that describes three specific scenarios: convergence, spatial variation, and stochastic variation, to define our hypotheses and expectations more clearly. The description of the convergence scenario is on lines 57-59. The spatial variation scenario is on lines:63-65. The stochastic variation scenario is on lines 67-70. These scenarios are also referred to at the end of the introduction (lines: 111-123) and the discussion (lines: 443-445, 462-465, 572-274). We believe this addition will make the introduction clearer and help the reader understand our expectations.***

Reviewer #2: (No Response)
